# Rheumatoid arthritis patients with peripheral blood cell reduction should be evaluated for latent Felty syndrome: A case report

**DOI:** 10.1097/MD.0000000000023608

**Published:** 2020-12-18

**Authors:** Peng Wu, Weifeng Sun, Jing Li

**Affiliations:** a The First Affiliated Hospital of GuangDong Pharmaceutical University; b Department of traditional Chinese medicine, southern theater general hospital, the Chinese People's Liberation Army, Guangzhou, China.

**Keywords:** Felty syndrome, glucocorticoid, peripheral blood cell, rheumatoid arthritis

## Abstract

**Rationale:**

Felty syndrome is a rare and life-threatening type of rheumatoid arthritis (RA).

**Patient concerns:**

A patient with RA had skin rash and subcutaneous hemorrhage, with a significant decrease in blood hemoglobin (Hb), white blood cell count (WBC), and blood platelet count (BPC).

**Diagnoses:**

The patient had a history of RA, splenomegaly, decreased Hb, WBC, BPC, and normal immunological indexes, combined with a series of bone marrow related tests and genetic tests.

**Interventions:**

She was given high-doses of glucocorticoids intravenously, followed by oral prednisone and cyclosporine maintenance therapy.

**Outcomes:**

Her symptoms were resolved within 2 weeks after the start of immunosuppression. After 2 weeks of discharge, the Hb, WBC, BPC basically returned to normal, and prednisone gradually decreased.

**Lessons:**

Felty syndrome is a rare complication of RA. Reductions in Hb, WBC, BPC, and subcutaneous hemorrhage should be considered strongly as the possibility of Felty syndrome. Multi-disciplinary diagnosis and related tests of bone marrow and genes are helpful for diagnosis and correct treatment.

## 1 Introduction

Felty syndrome is a rare and special type of rheumatoid arthritis (RA) that is typically characterized by arthritis, neutropenia, and splenomegaly. Common symptoms include systemic lymphadenopathy, anemia, thrombocytopenia, and lower-extremity ulcers.^[13]^ Latent Felty syndrome has a higher mortality rate than RA because clinicians cannot detect the leukopenia in time, leading to delayed treatment. Related studies have shown that Felty syndrome is common in patients with a long history of RA (>10 years). However, for patients with a short RA history and no clinical signs or symptoms, Felty syndrome is often easily missed or misdiagnosed, leading to delayed treatment.^[5,14,19]^ Therefore, here, we report the case of a patient with a short history of RA (<1 year) that presented as subcutaneous hemorrhage and decreased hemoglobin (Hb), WBC, and blood platelet count (BPC). After the exclusion of other systemic diseases, the final diagnosis was Felty syndrome. Treatment with glucocorticoids and immunosuppressive agents reduced the peripheral blood cell count and healed the skin.

## 2 Case presentation

The study was approved by the Human Body Protection Agency Review Board of the Southern Theater General Hospital and followed the principles of the Helsinki Declaration. The patient provided written informed consent and has agreed to publish the case.

In December 2017, a 48-year-old woman developed repeated pain in the thumb and metacarpophalangeal joints, forefinger metacarpophalangeal joints, proximal interphalangeal joints, bilateral wrist joints, and bilateral shoulder joints with a morning stiffness of 1.5 h. Pain symptoms were relieved after symptomatic treatment in the hospital. In April 2018, she visited our department. Laboratory values were as follows: anti-CCP antibody, 401.00 U/mL; erythrocyte sedimentation rate, 40.00 mm/h; C-reactive protein, 6.7 mg/L; rheumatoid factor, negative; anti-RA33 antibody, negative; and anti-“O” test, negative. She was subsequently diagnosed with RA. Treatment with meloxicam dispersible tablets 7.5 mg/day, methotrexate (MTX) 10 mg/week, leflunomide (LEF) 20 mg/night, prednisone 7.5 mg/day at 8 am for 2 weeks alleviated her symptoms of joint swelling and pain. However, she reduced the medication amount due to stomach discomfort. Three days prior to presenting, the patient developed a red rash on the chest, abdomen, and back that partially merged into an area with obvious itchiness. A rash then developed on the hips and feet. Later, subcutaneous sputum and ecchymoses appeared on the lower extremities, particularly the bilateral calves. A small amount of petechia appeared on the upper limbs without pain, itching and bleeding gums, at which point she visited our outpatient clinic.

**Figure 1:**
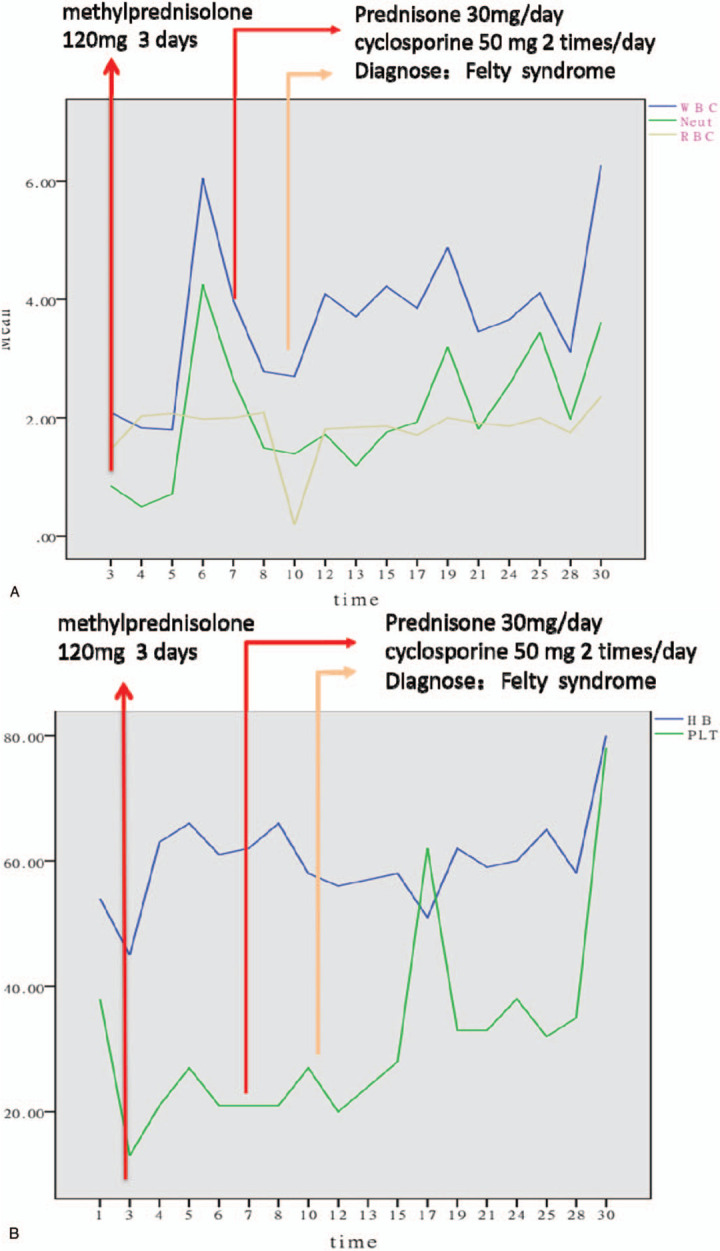
Diagnosis and treatment progress of the patient. (A) Neut = neutrophils, RBC = red blood cells, WBC = white blood cell. (B) Hb = blood hemoglobin, PLT = platelet.

**Figure 2:**
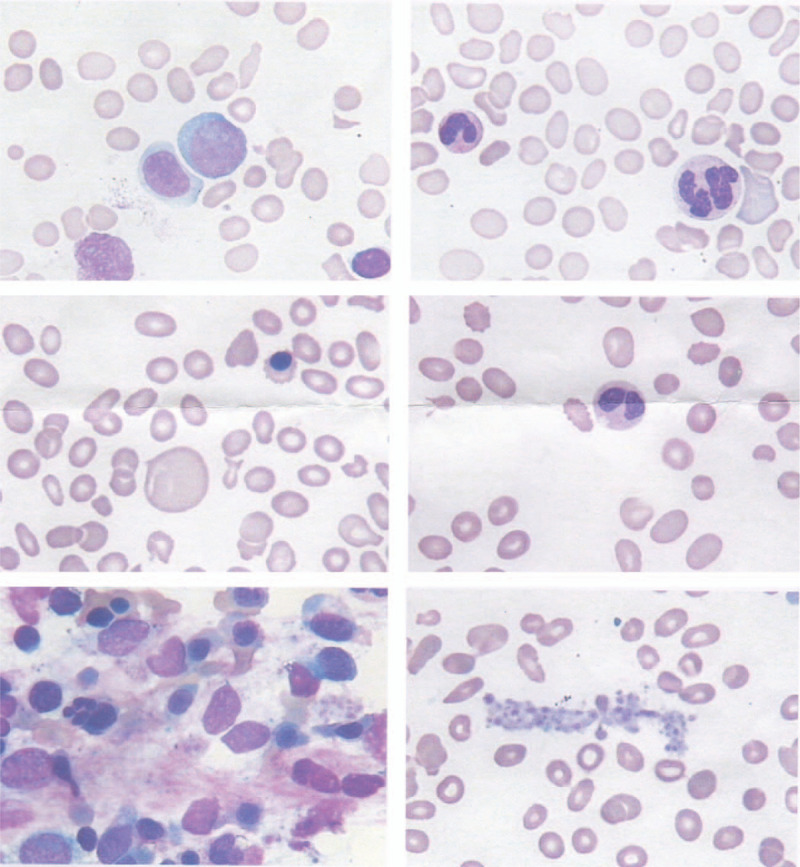
Bone marrow aspiration results (day 4 bone marrow aspiration).

The patient's vital signs were stable at admission, but routine bloodwork showed the following: WBC, 2.09 × 10^9^/L; neutrophils, 0.85 × 10^9^/L; red blood cells, 1.46 × 10^12^/L; Hb, 45 g/L, BPC, 13 × 10^9^/L ↓ (Table 1). Thus, we discontinued all drugs. After the dermatology consultation, drug dermatitis, and purpura were considered, and she was given desonide ointment and ebastine tablets for anti-allergy treatment. A hematology consultation determined that blood system diseases could not be ruled out. A bone marrow puncture was recommended. Due to the patient's critical situation, symptomatic treatment including the infusion of 2 units of red blood cells and 15 units of platelets and a methylprednisolone 120 mg intravenous drip for 3 days were given (Fig. 1). On the fourth day of admission, the lower-extremity subcutaneous ecchymosis decreased, the rash on the chest and abdomen faded significantly, the back and foot rashes were slightly better, and the WBC, Hb, and BPC values were significantly improved (Table 1). The relevant examinations continued. The Coombs test, electrolyte, and total biochemical findings were normal. The fibrinogen was 4.5 g/L (reference value, 2–4 g/L), while partial clotting activity enzyme time was 45.2 s (reference value, 28–44 s). The rheumatoid immune index results showed no obvious abnormalities (Table 2). The bone marrow puncture results showed that myelodysplastic syndrome (MDS) should be considered (Fig. 2). An abdominal B-ultrasound showed that the spleen was ∼158 × 47 mm and the rib was about 49 mm. The liver, gallbladder, pancreas, kidneys, and so on showed no obvious abnormalities. On the seventh day after admission, the patient developed a systemic rash and significantly reduced WBC, Hb, and BPC values (Table 1). A bone marrow flow test showed 0.15% normal myeloid naive cells in the bone marrow samples and abnormal granulocyte differentiation and development; thus, MDS was not excluded. The genetic examination revealed three chromosomal abnormalities. A database search and literature review confirmed that the three abnormalities were not related to the pathological changes associated with malignant blood diseases. Gain (14q) is included in the normal population chromosome copy number variation polymorphism database, while two uniparental disomies (Xq) were included in the normal population uniparental disomy database. Analysis of bone marrow karyotype showed that this patient's specimen was analyzed for 2 metaphase cells after culture. No abnormal chromosome clones were found. Because few metaphase cells were available for analysis, review of the elective period recommended. After the temporary administration of granulocyte colony-stimulating factor as whitening treatment and the administration of prednisone 30 mg/day and cyclosporine 50 mg 2 times/day, the patient's rash and sputum point subsided significantly. On the 10th day, the WBC, Hb, and BPC steadily increased (Table 1). Combined with the patient's clinical manifestations and related test results, MDS was excluded and Felty syndrome was diagnosed (Fig. 1). The treatment of prednisone 30 mg/day, cyclosporine 50 mg 2 times/day was continued. On the 10th day, the peripheral blood cell count continued to rise steadily and the patient was discharged on the 15th day. A reexamination 2 weeks after the patient was discharged from the hospital showed the WBC, Hb, and BPC were basically normal, so the prednisone was gradually reduced and the outpatient treatment was continued.

**Table 1:** Patient's laboratory test results during hospitalization and follow-up (abnormal values in bold).

Blood test	Admission	Day 4	Day 7	Day 10	Day 15	2 week after discharge
WBC (4.0–10.0) × 10^9^/L	**2.09**	6.05	**2.7**	**3.85**	6.27	6.74
Neutrophils % (0.5–0.7)	0.85	4.25	1.39	1.93	3.62	3.74
RBC (3.5–5.0 × 10^12^/L)	**1.46**	**1.98**	**0.19**	**1.71**	**2.36**	3.74
Hb (110–150 g/L)	**45**	**61**	**58**	**51**	**80**	111
PLT (100–300) × 10^9^/L)	**13**	**21**	**27**	**62**	**78**	120
Scr (44–97 umol/L)	47	48	49	46	43	45
BUN (3.2–7.1 mmol/L)	**1.9**	3.2	3.5	3.2	3.6	3.3
ALT (0–40 U/L)	20	23	22	25	29	24
AST (0–40 U/L)	27	23	25	23	26	25
LDH (100–300 U/L)	243	249	265	243	253	233
Sugar (3.9–6.1 mmol/L)	5.5	5.8	4.9	4.8	5.2	4.3
ALB (35–51 g/L)	41	38	39	42	43	44
UPQ (≤150 mg/24 h)	0.10	0.18	0.04	0.02	0.03	0.06
uric acid (89–357 μmol/L)	325	300	329	276	289	342
K (3.5–5.5 mmol/L)	3.7	3.5	3.6	3.7	3.9	4.4
Na (135–145 mmol/L)	139	138	140	141	138	140
Cl (96.00–106.00 mmol/L)	105	102	98	101	99	103
ESR (0–20 mm/h)	**22**	15	14	16	10	8
CRP (0.068–8.2 mg/L)	**59.8**	**25**	6	3	5	4
Fg (2–4g/L)	**4.5**	3.8	3.2	3.5	3.7	3.2
PTT (28.00–44.00 S)	**45.2**	43	40	43	39	42
Procalcitonin (<0.5 ug/L)	0.08	0.04	0.03	0.02	0.04	0.02

**Table 2:** Patient's immunological data.

Name	Immunological data
C3 (0.80–1.20 g/L)	1.01
C4 (0.44–0.66 g/L)	**0.275**
IgG (6–16 g/L)	**8.14**
IgM (40–345 mg/dL)	**0.45**
IgA ((76–390 mg/dL)	**1.85**
Anti-Un Ab (<1:160)	4.82
Anti-ds-DNA Ab (negative)	Negative
Anti-Sm Ab (negative)	Negative
Anti-SSA Ab (negative)	Negative
Anti-SSB Ab (negative)	Negative
Anti-caIgM (negative)	Negative
Anti-caIgG (negative)	Negative
Anti-ma Ab (negative)	Negative
AGBM Ab (negative)	Negative
Anti-p3 Ab (negative)	Negative
Anti-B2IgG (negative)	Negative
Anti-B2IgM (negative)	Negative

## 3 Discussion

Felty syndrome is common in patients with a history of RA longer than 10 years. For those patients with a short onset time and atypical clinical symptoms, a missed or incorrect diagnosis is not uncommon. Therefore, early diagnosis is of great significance for the treatment and prognosis of Felty syndrome.^[18]^ Felty syndrome can be confused with immune system diseases like systemic lupus erythematosus (SLE) and Sicca syndrome (SS), blood system diseases like MDS and aplastic anemia (AA), digestive system diseases such as cirrhosis and adverse drug reactions.^[9,18]^

In this case, the patient developed a decreasing peripheral blood cell count and some skin symptoms. Upon admission, the cause of the peripheral blood cell reduction was unclear since drugs or SLE, MDS, AA, or other diseases may cause such decreases. However, in each case, it can be treated with glucocorticoids, so methylprednisolone 120 mg was given to treat it.^[11]^ Since the disease onset, there were no photoallergic symptoms, nervous system involvement, oral ulcers, kidney damage, dry eyes, dry mouth, caries, repeated parotid swelling, or other symptoms. Although reduced WBC, Hb, and BPC were seen, no hemolytic anemia was noted. The laboratory results showed that Coomb's test was negative, while a rheumatoid immunological examination showed no obvious abnormalities, thereby excluding other rheumatoid immune system diseases such as SLE and SS.^[12]^ Because the patient had a history of RA, the possibility of Felty syndrome was considered. Blood system diseases such as MDS and AA can decrease peripheral blood cell counts. Therefore, bone marrow puncture is of great significance for the differential diagnosis. In this case, the patient underwent two bone marrow punctures, and it was not ruled out that the decrease in the blood triads was caused by MDS, so further tests like bone marrow biopsy, bone marrow flow, bone marrow chromosome, and genetic examinations are necessary.^[12,24]^ Since the patient refused to undergo a bone marrow biopsy, only bone marrow flow, bone marrow chromosome, and genetic examinations were performed. The results suggested that it was unlikely to have been caused by MDS. After a multi-disciplinary consultation, doctors believed that MDS could not explain the splenomegaly, so malignant hematological diseases such as MDS and AA are excluded, making the diagnosis more likely Felty syndrome. MTX can cause many adverse reactions, including reduced WBC, Hb, and BPC, but the resulting myelosuppression is reversible.^[8]^ The most common adverse reactions of LEF include digestive tract reaction, rash, hair loss, weight loss, and liver and kidney dysfunction but rarely decreases WBC or platelet blood count.^[10]^ This patient had a short-term (2-week) history of MTX and LEF use and experienced gastrointestinal adverse reactions. She subsequently reduced the dosage herself and experienced a series of signs including rash and subcutaneous ecchymosis. Combined with splenomegaly visualized on abdominal B-ultrasound, lack of portal hypertension, and normal liver and kidney function, it is unlikely that the decrease in WBC, Hb, and BPC was caused by adverse drug reactions. After the exclusion of these diseases, Felty syndrome was diagnosed and treated.

The cause of the peripheral blood cell reduction in Felty syndrome is not fully understood. Neutropenia, the most common symptom, may be related to the presence of granulocyte-specific anti-nuclear factor (GS-ANF). The positive rate of GS-ANF in patients with Felty syndrome is reportedly as high as 75% to 100%, while that in RA patients is only 25% to 30%.^[16,20]^ At the same time, the presence of IgG-like granulocyte antibodies in peripheral blood of patients with Felty syndrome can further destroy granulocytes, decreasing their ability to phagocytose immune complexes, while T-cell activation can inhibit granulocyte production. Similar to RA patients, anemia is common in patients with Felty syndrome. Splenomegaly can cause thrombocytopenia, the mechanism of which may be related to factors like decreased platelet production, spleen retention, peripheral platelet depletion, and peripheral immune-mediated platelet destruction.^[2]^

A special type of RA, Felty syndrome is treated similarly to RA, with the treatment being mainly divided into glucocorticoids and slow-acting anti-rheumatic drugs. However, due to the lack of evidence-based medicine, most are empirical drugs.^[13,18]^ Glucocorticoids are widely used to treat Felty syndrome because of their strong anti-inflammatory and immunosuppressive effects. As a traditional drug for patients with significantly reduced WBC, Hb, and BPC, large doses of hormones combined with gamma-globulin can be given.^[11]^ In this case, gamma-globulin shock therapy was rejected due to the patient's economic level. After the condition stabilizes, the glucocorticoids should be gradually reduced. Immunosuppressive drugs can be used to treat Felty syndrome. Both MTX and LEF can improve joint and vascular inflammation in patients with Felty syndrome.^[4,26]^ Sulfasalazine suppositories can directly inhibit or even kill the activated immunoactive cells, and they have positive impacts on pain relief and disease control.^[3]^ Some clinical trial results have shown that cyclophosphamide effectively treats Felty syndrome, but the resulting reduction in granulocytes limits its use.^[15]^ However, the significant reductions in WBC, Hb, and BPC limit the use of immunosuppressive agents. As a second-line drug, cyclosporine inhibits T-cell activation by inhibiting calcineurin and can be used in patients with blood system diseases such as leukopenia and thrombocytopenia.^[1,17]^ Therefore, in our case, cyclosporine was administered. The peripheral blood cell count returned to normal after 2 weeks of follow-up, while the skin lesion symptoms completely disappeared.

In recent years, biological agents have been widely used to treat various rheumatic immune diseases. Currently, the most widely used agent is rituximab (RTX), a monoclonal antibody to CD20 that can fight mature B cells and has been approved for the treatment of complex RA. Related studies have shown that RTX can be used to successfully treat Felty syndrome with infection or ulceration.^[5,13,23]^ Among them, the use of RTX to reduce WBC, Hb, and BPC remains controversial. Lekharaju, Fragoso, and other authors^[6,7,20,21,25]^ indicated that RTX can successfully reverse the decreased WBC and neutropenia in patients with Felty syndrome, while reports by Sordet C and Salama^[20,22]^ were unable to conclude that RTX can successfully reverse WBC reductions and neutropenia in patients with Felty syndrome. However, the overall results demonstrated that RTX still has a high successful rate for treating refractory Felty syndrome.

We reported here the case of a patient with a short-term (<1 year) duration of LA plus latent Felty syndrome with a reduced peripheral blood cell count and skin symptoms. The main purpose of this study was to emphasize that, when the reduction of peripheral blood cell count in the absence of the common symptoms of RA such as arthritis occurs, the possibility of Felty syndrome should be considered and the diagnosis confirmed quickly. Active intervention would positively affect patient prognosis. We can gain some experience and inspiration from this case. First of all, if blood counts decrease in a case of newly developed RA, clinicians should be alert to the possibility of latent Felty syndrome. Second, bone marrow puncture is recommended in routine examinations to rule out blood system diseases. Third, multidisciplinary communication helps with the early diagnosis of Felty syndrome. Fourth, for the treatment of Felty syndrome according to the patient's condition, active glucocorticoids and gamma-globulin shocks are beneficial to controlling the disease. Fifth, patients in whom conventional immunosuppressive agents and glucocorticoids are ineffective should be actively treated with biological agents such as RTX.

## Author contributions

**Conceptualization:** Weifeng Sun.

**Writing – original draft:** Peng Wu.

**Writing – review & editing:** Peng Wu, Jing Li.
